# Supplementary material for: Spatial self-organization resolves conflicts between individuality and collective migration
Source: Nat Commun. 2018 Jun 5;9:2177. doi: 10.1038/s41467-018-04539-4 (PMC5988668; doi:10.1038/s41467-018-04539-4)
Supplement: Supplementary file 1 — Supplementary Information [file 41467_2018_4539_MOESM1_ESM.pdf]

Supplementary Information

Spatial Self-Organization Resolves Conflicts Between Individuality and Collective Migration

X. Fu, S. Kato, J. Long, H.H. Mattingly, C. He, D.C. Vural, S.W. Zucker and T. Emonet

This file contains Supplementary Figures 1-9 and Supplementary References.

Content

Supplementary Figures 1

Supplementary Figure 1 | Schematic of the experiment........ 1

Supplementary Figure 2 | Labeling cells with RFP1 or YFP and dilution process does not affect tumble bias distribution nor band speed. 2

Supplementary Figure 3 | Dilution process does not affect the distribution of tumble biases. ... 3

Supplementary Figure 4 | Doubling time in the device is longer than the duration of the experiment. 4

Supplementary Figure 5 | Calculation of speed, cell density, and number of cells in the traveling band. 5

Supplementary Figure 6 | Aspartate dominates the chemotaxis response. 7

Supplementary Figure 7 | Aspartate consumption depends on oxygen which becomes limited in the center of the traveling band. 9

Supplementary Figure 8 | Model details and the effect of the oxygen-dependent aspartate consumption on cell leakage........ 11

Supplementary Figure 9 | Spontaneous ordering of phenotypes in a traveling band. 12

Supplementary References 13

Supplementary Figures

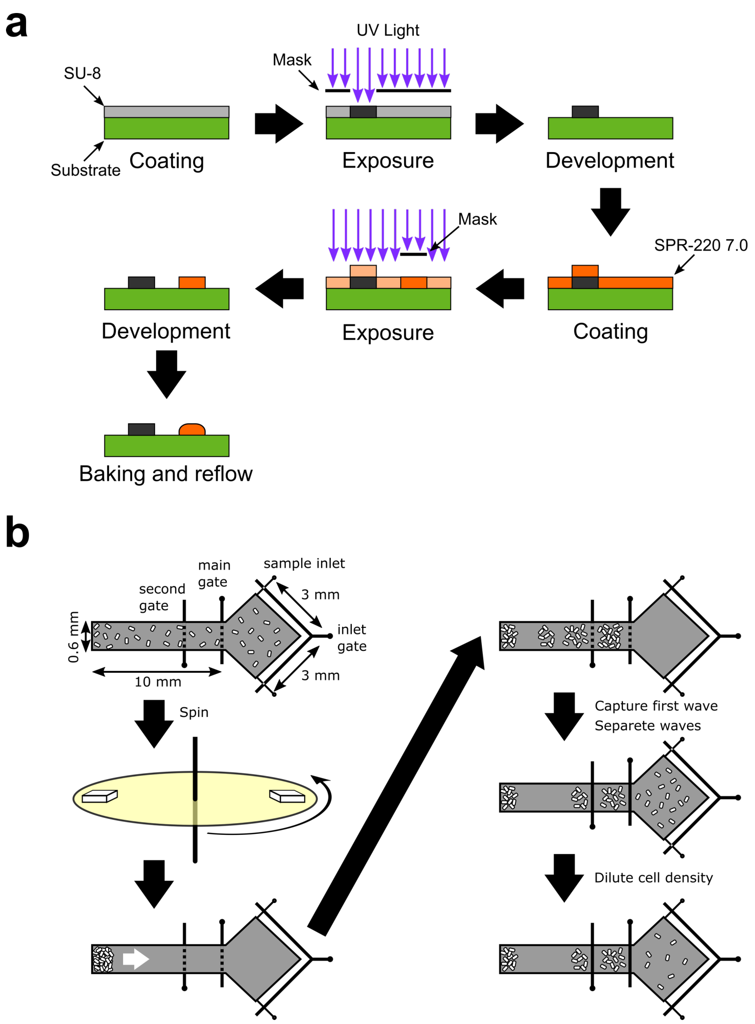

Supplementary Figure 1 | Schematic of the experiment.
a. Schematic of micro fabrication process of the mold for the bottom layer of the Y shape microfluidic device. The mold is made from two types of photoresists, MICROCHEM SU8-3010 and MEGAPOSIT SPR 220-7.0, with a standard micro fabrication protocol. The SPR 220-7.0 is used so as to achieve a 14 μm height channel with a rounded ceiling by reflowing. The rounded ceiling ensures that the control valves on the top layer close the channel tightly by pressure. **b**, Schematic of the experiment. After loading cells everywhere, the device was spun for 20 minutes at 700 g on a 15 cm petri plate, resulting in the accumulation of cells at the one end of the channel. Following spinning, cells immediately started to migrate. First and second bands were separated using control gates. After capturing a band of cells, fresh media was perfused into the observation chamber (diamond) to remove attractant and reduce cell density. Swimming trajectories were recorded to extract individual tumble bias. We verified that perfusing the chamber to lower cell density did not significantly affect the tumble bias distribution (Supplementary Fig. 3).

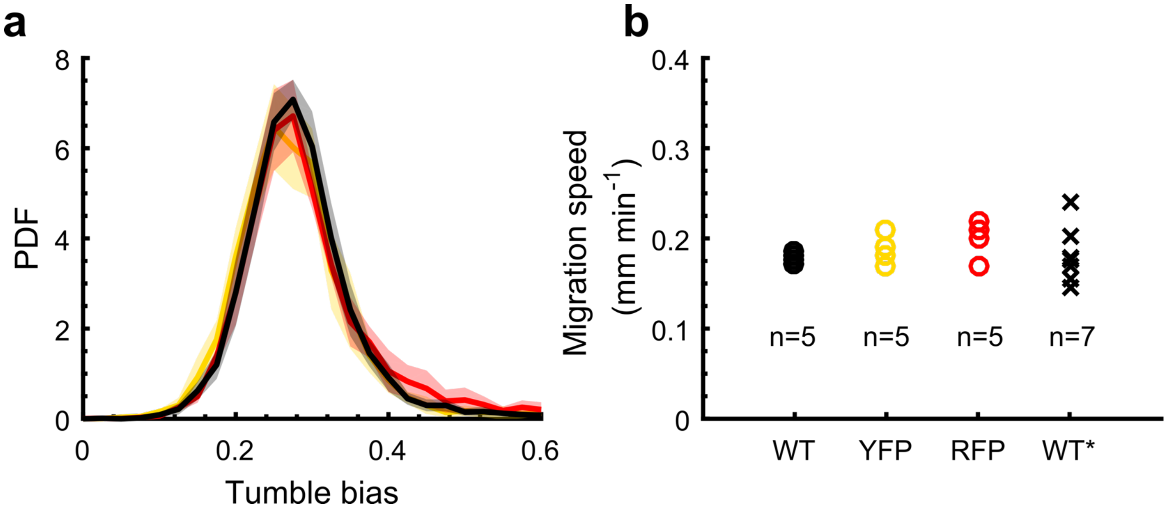

Supplementary Figure 2 | Labeling cells with RFP1 or YFP and dilution process does not affect tumble bias distribution nor band speed.

a. Tumble bias distributions of the wild type (RP437) strain used throughout this study (black) and of the same strain transformed to express mRFP1 (red) or YFP (yellow) for cells grown in M9 glycerol medium (M9 salts, glycerol and casamino acids; Methods). Cells were sampled at ~OD600 0.3. One sample from the culture was placed in diluted M9 glycerol medium between a glass slide and a coverslip and individual cells were tracked to measure the TB distribution. Line: mean with n>4; shading: standard deviation. Another sample from the same culture was used in the microfluidic device to measure collective migration speed. **b**, Migration speeds of the three strains when migrating in the presence of 200 μM Aspartate. All experiments were performed in the 14 μm-deep microfluidics chamber with the exception of WT*, which was done in 100 μm-deep chamber, indicating the depth of the chamber doesn't affect the migration speed. Number of replicate experiments is shown in the figure.

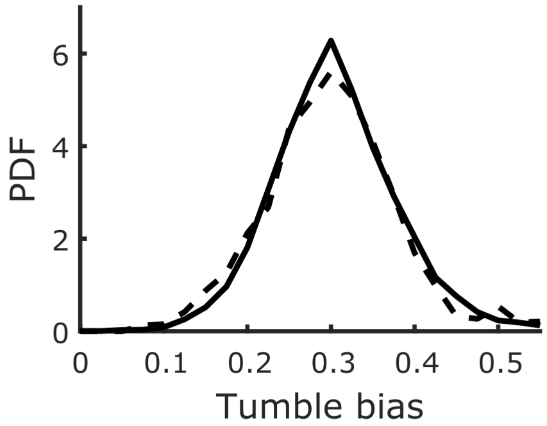

Supplementary Figure 3 | Dilution process does not affect the distribution of tumble biases.

Wild type cells mixed with mRFP1 fluorescence-labelled cells (in M9 glycerol medium, OD600~0.7, ratio ~20:1) were loaded into the microfluidics device via inlet hole using a syringe. Time lapse movies of the chamber were recorded using RFP filter set while the inlet/outlet valves and the right control gate were closed, and the tumble bias distribution was analyzed (dashed line). Then, fresh medium was perfused into the device to dilute the cell density down to an OD600 of ~0.03-0.08. Phase contrast time lapse movies were then recorded to obtain swimming behavior after dilution (solid line).

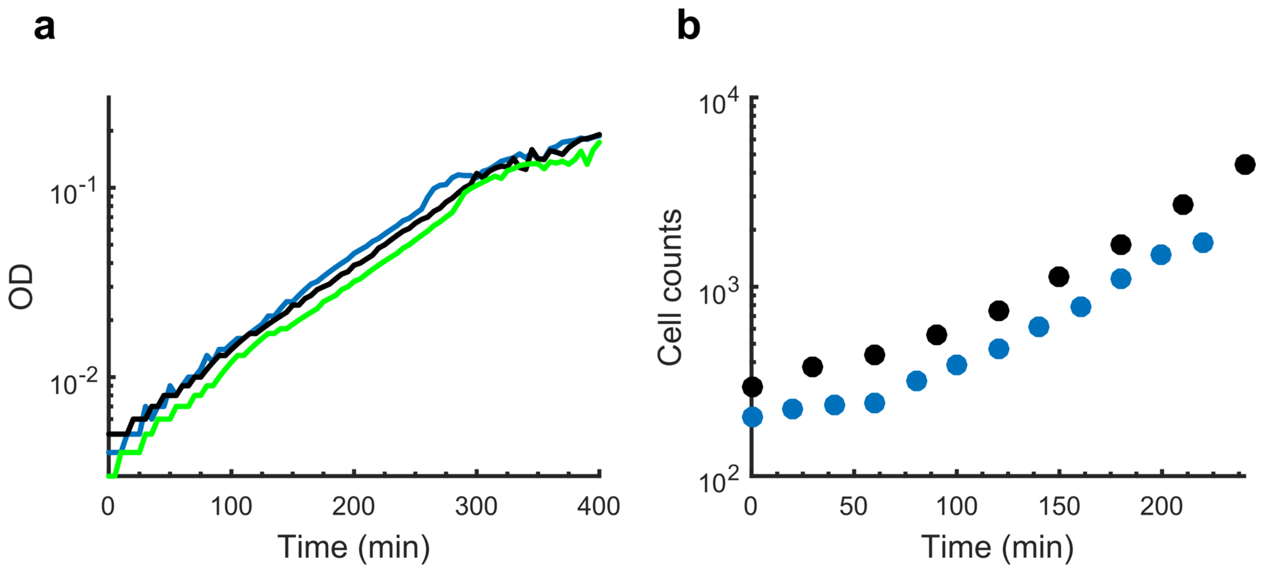

Supplementary Figure 4 | Doubling time in the device is longer than the duration of the experiment.

a, Growth curves of RP437 in 96 well microplates. Colors: biological replicates. Doubling times: 60.1 ± 1.1 min (blue), 66.2 ± 0.6 min (black), and 62.9 ± 1.2 min (green). **b,** Growth curves in the observation chamber of the microfluidics. Colors: biological replicates. Doubling times: 55.6 ± 3.8 min (blue) and 53.4 ± 7.1 min (black). A low density cell culture with fresh M9 glycerol medium was loaded into the device. Every 30 minutes, 2 minute (8.2 fps) movies were recorded at four different locations to cover the entire observation chamber. For each movie, we calculated the average number of cells over 984 frames. The total number of cells was calculated by summing up cell numbers at all four locations.

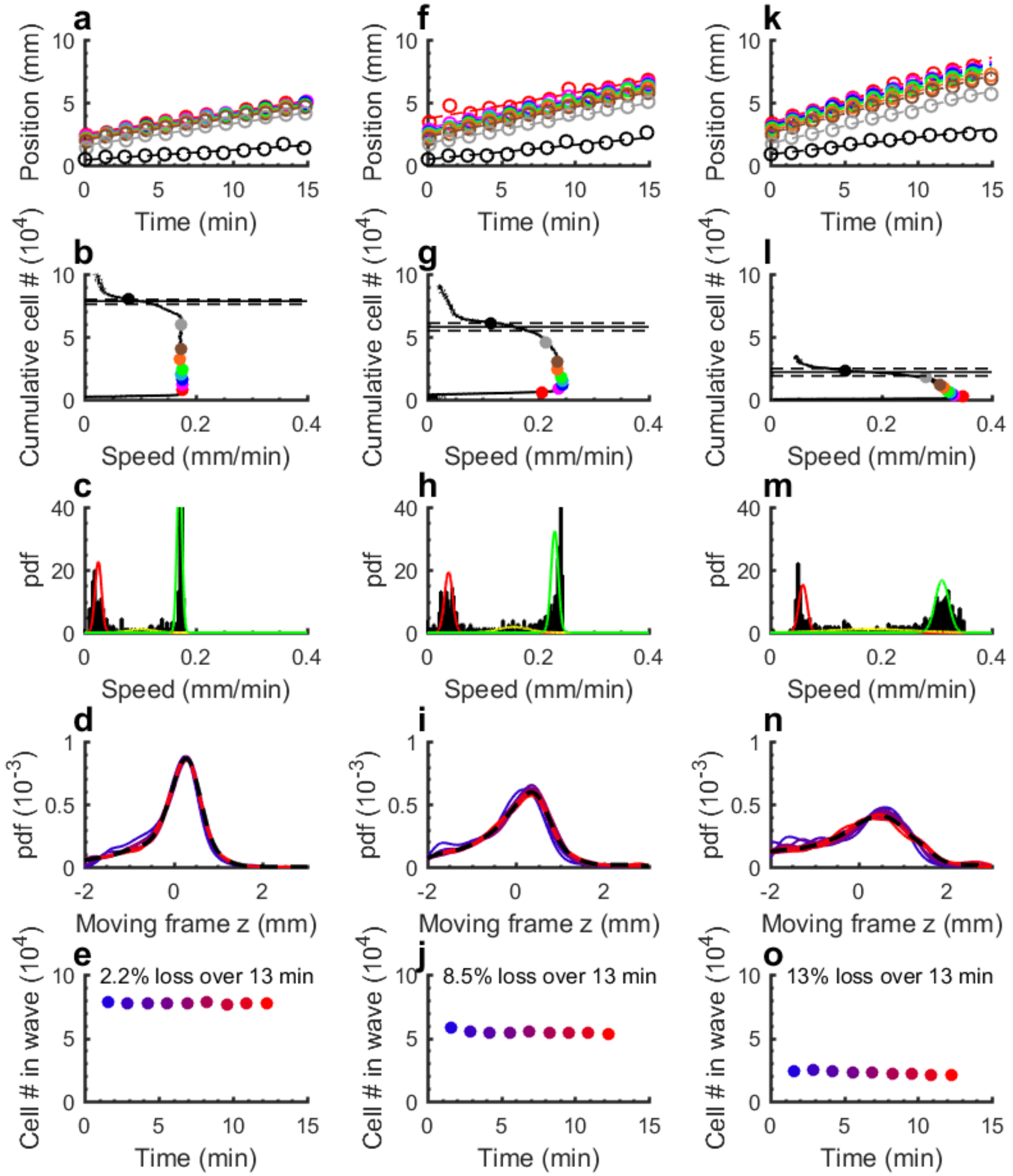

Supplementary Figure 5 | Calculation of speed, cell density, and number of cells in the traveling band.

a, Position of the n'th cell (counted from the front of the band) as a function of time (circle) and their linear fits (dashed) in 200 μM aspartate (time intervals between consecutive images in a sweep of the channel $\Delta t = 3$ s, and bin size within which cells are counted $\Delta x = 122$ μm). Colors indicate values of n. Uncertainties are too small to show. **b,** Traveling speed as a function of n. Black solid curve: slope of the linear fit in panel **a**. Black dotted curve: standard error in slope of

the fit. Filled colored circles indicate values of n shown in panel **a**. Horizontal solid and dashed lines indicate the number of cells in the wave and its uncertainty as determined in panel **c**. **c,** The pdf histogram of the speeds in panel **b** and a 3-component Gaussian mixture fit. The 3-components of Gaussian distributions of speeds indicate cells that are traveling (green), falling off (yellow), and staying behind (red). We use the mean and standard deviation of the green Gaussian as the wave speed and its uncertainty. We estimate the number of cells in the wave as the value of n in panel **b** where the speed drops to the mean speed of the yellow Gaussian in panel **c**. **d,** Cell density pdf's in the wave, as determined in **b**, at 9 time points in 1.3-minute intervals. The last one is when the fastest wave approached the end of the channel. Colors corresponding to time in panel **e**. Dashed: weighted average of cell density pdfs over time, with weight given by distance to the mean pdf. Pdf's were estimated using kernel density estimation with a Gaussian kernel of bandwidth 200 μm (> bin size 122 μm). **e,** Number of cells in the wave as a function of time. **f-j,** and **k-o** same as **a-e** but for bands traveling in 100 and 50 μM aspartate, respectively.

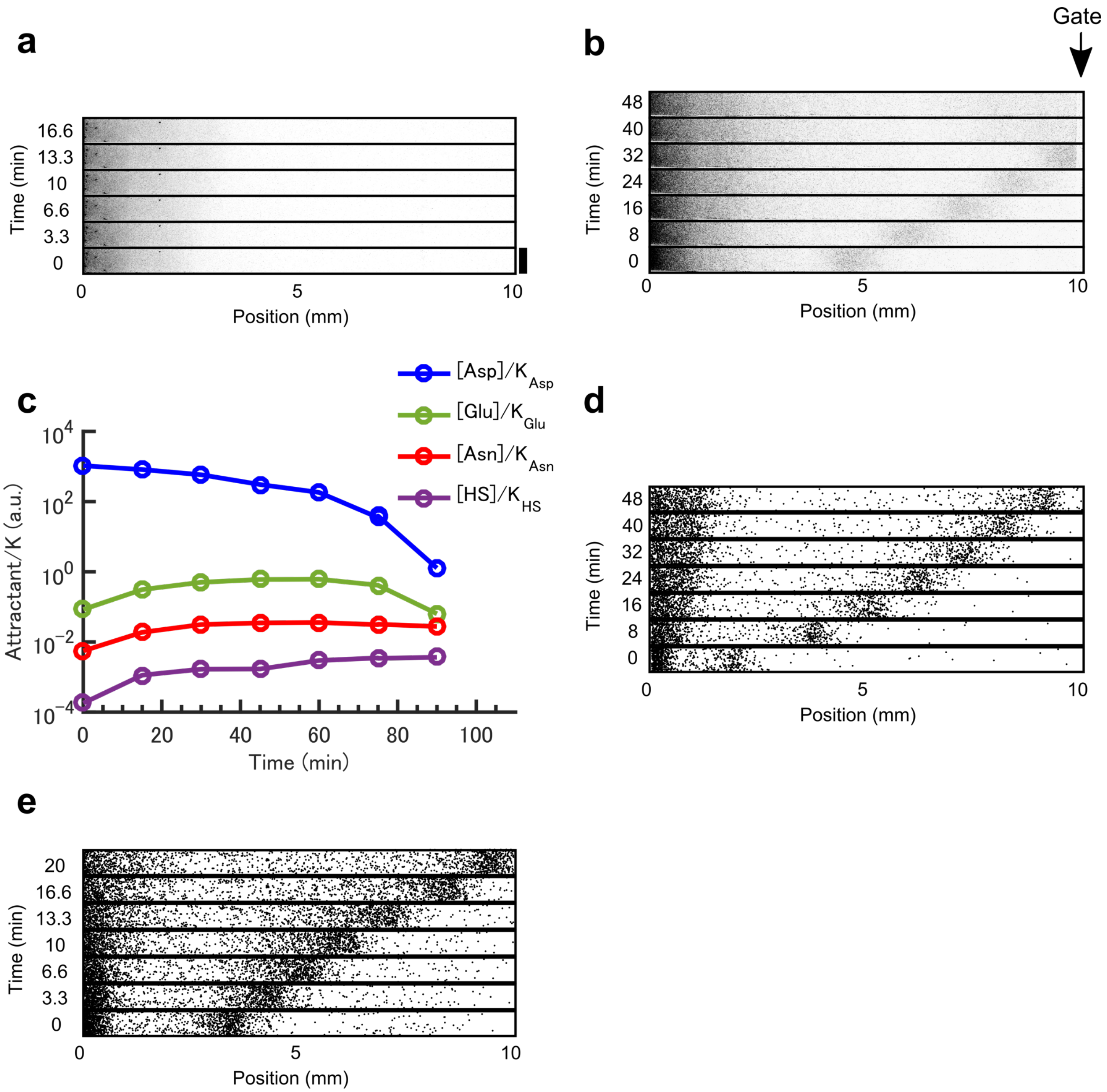

Supplementary Figure 6 | Aspartate dominates the chemotaxis response.

a, Time-lapsed fluorescent images of cells expressing YFP in M9 glycerol buffer without supplemented aspartate. No band emerged, cells are just diffusing. Scale bar, 0.6 mm. **b,** Same with 200 μM aspartate supplemented and a closed gate ahead located at 10 mm. After reaching the closed gate the band stayed near the gate and diffused but did not migrate backwards as a band, suggesting that all aspartate had been consumed. Together (**a**) and (**b**) indicate that under these conditions no consumable attractant other than aspartate is available to drive the band. **c,** Amino acids present in M9 glycerol buffer supplemented with 500 μM aspartate as a function of time (Methods), plotted in units of the corresponding K_0 of the dose response of the chemotaxis system for each amino acid (EC_{50} of RP437 *E. coli* is 0.3 μM for aspartate (Asp), 50 μM for glutamate (Glu), 30 μM for asparagine (Asn), and 3 mM for homoserine (HS)[1, 2]). Upon uptake of aspartate, cells secreted small amounts of attractant amino acids. However, at these

concentrations the chemotactic response to aspartate would be approximately one thousand times stronger than the response to the other amino acids. **d and e,** Aerotaxis does not play a significant role. Mutant strains lacking the oxygen receptor Aer (**d**) or Aer and Tsr (**e**) form a traveling band under the same conditions as wt cells in Fig. 2a. A 1:200 and 1:100 dilution of fluorescently-labeled cells was used, respectively.

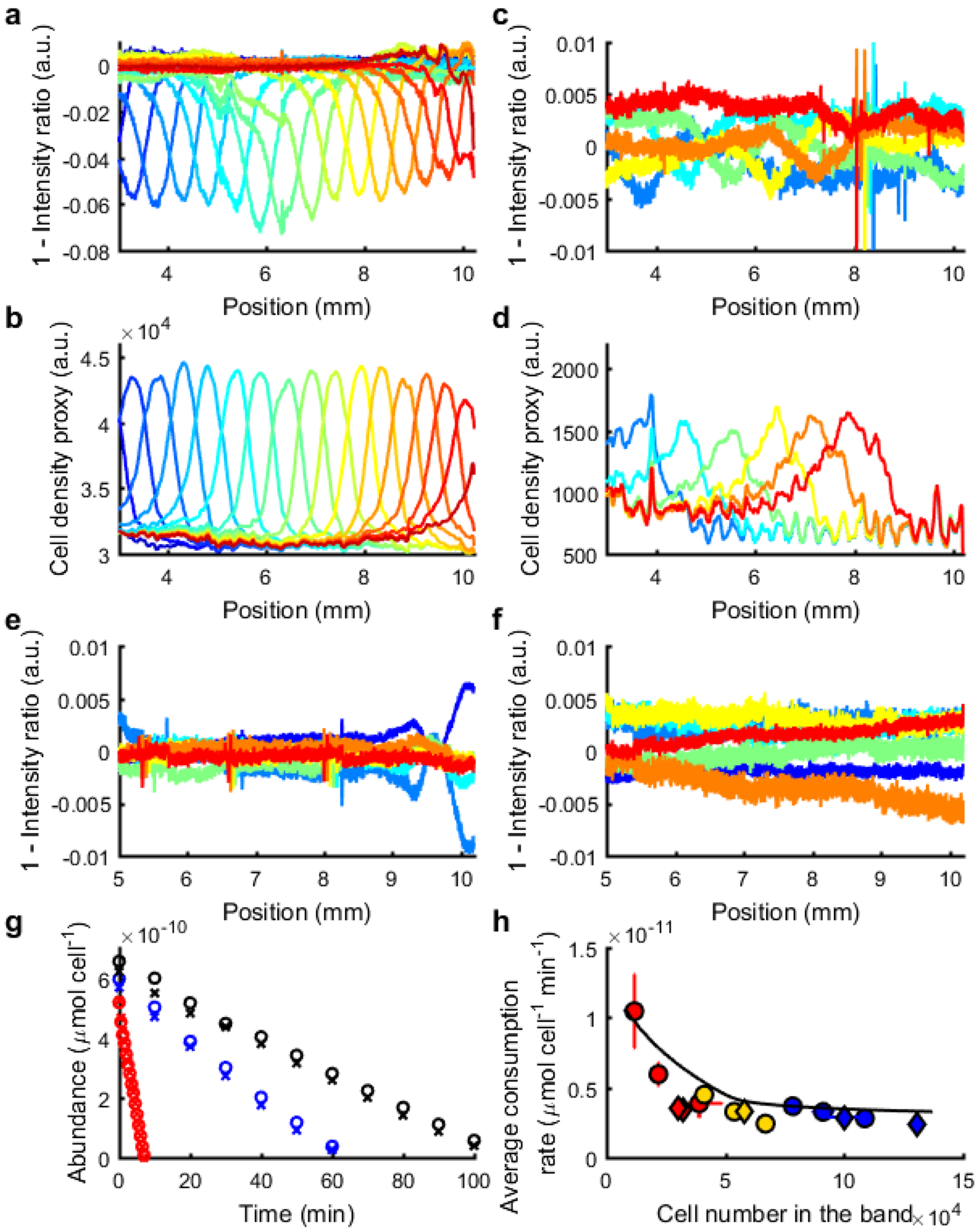

Supplementary Figure 7 | Aspartate consumption depends on oxygen which becomes limited in the center of the traveling band.

a, In a 100 µm-deep microfluidic device, signal from the ruthenium complex oxygen sensor coincides with the traveling wave in panel **b** (see Methods). Fluorescence of the sensor is

quenched by oxygen; therefore, 1 minus the normalized intensity is shown in all cases. Note that the signal is a nonlinear function of the oxygen concentration. **b,** A proxy for cell density for the experiment shown in panel **a** (average intensity of the phase images over the width of the channel). In panels **a** and **b**, colors from blue to red correspond to 4 through 38 minutes past the start of imaging, in 2-minute intervals. **c, d,** Same as **a, b** but in a 14 μm-deep device (see Methods). Here, the proxy for cell density is the standard deviation of intensity in the transmitted light image over the width of the channel. Signal from the oxygen sensor in **c** coincides with the traveling wave in panel **e.** Normalized fluorescence intensity in a 14 μm-deep device with dye but no cells, showing no wave structure. **f,** Normalized fluorescence intensity in a 14 μm-deep device with cells but no dye, also showing no wave structure. Red to blue in panels **c-f** are 1 through 23 minutes after the start of imaging, in 3.5-minute intervals. Data in panels **a**, **c**, **e**, and **f** were all processed the same way, as described in the Methods. The spread in panels **e** and **f** provide the noise scale in **c**. **g,** Red: Oxygen consumption (see Methods). Blue: Aspartate consumption with oxygen (see Methods). Black: Aspartate consumption without oxygen (see Methods). Circles and crosses are replicate experiments. **h,** Average consumption rate of aspartate in the band depends on cell density. Black: model prediction. Symbols: experimental estimates using Eq. 4 of the Methods (red: 50 μM; yellow 100 μM; blue: 200 μM; circle and diamonds are the same as in Fig. 2; error bars: standard deviation.)

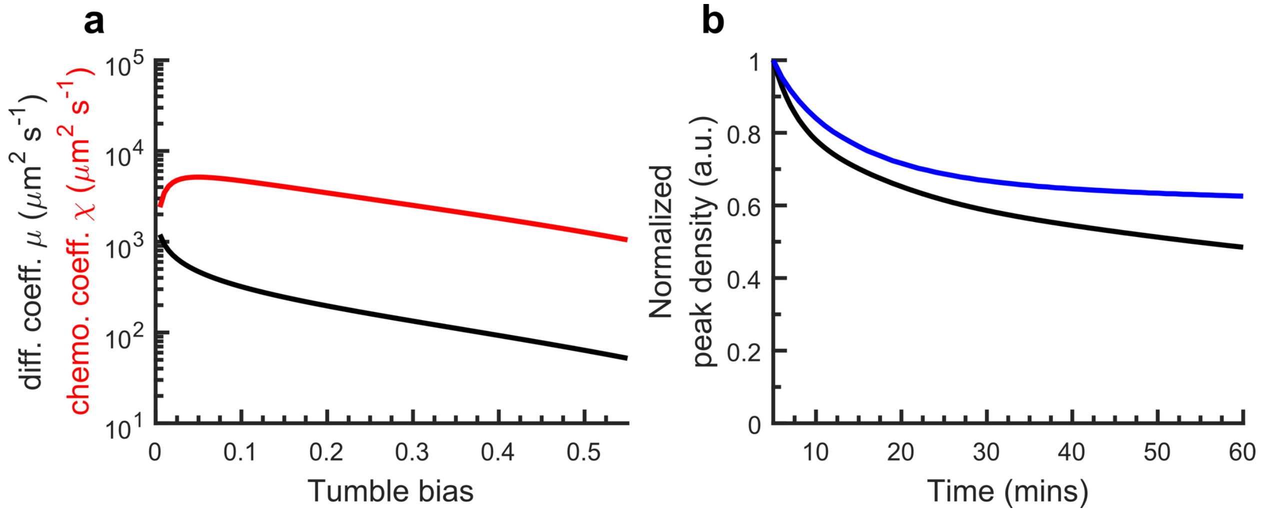

Supplementary Figure 8 | Model details and the effect of the oxygen-dependent aspartate consumption on cell leakage.

a, Diffusion coefficient μ (black) and chemotactic coefficient χ (red) as a function of tumble bias. **b,** Fraction of cells remaining in the wave relative to the number of cells at t=30 mins over time for two different simulations. Blue: oxygen-dependent aspartate consumption rate (same simulation as in Fig 2 (dashed blue)). Black: constant aspartate consumption rate (here we used $\frac{2}{5}\alpha_{A0}$ to get a wave speed similar to the experiment). See Methods and Table 1 in the main text for parameters.

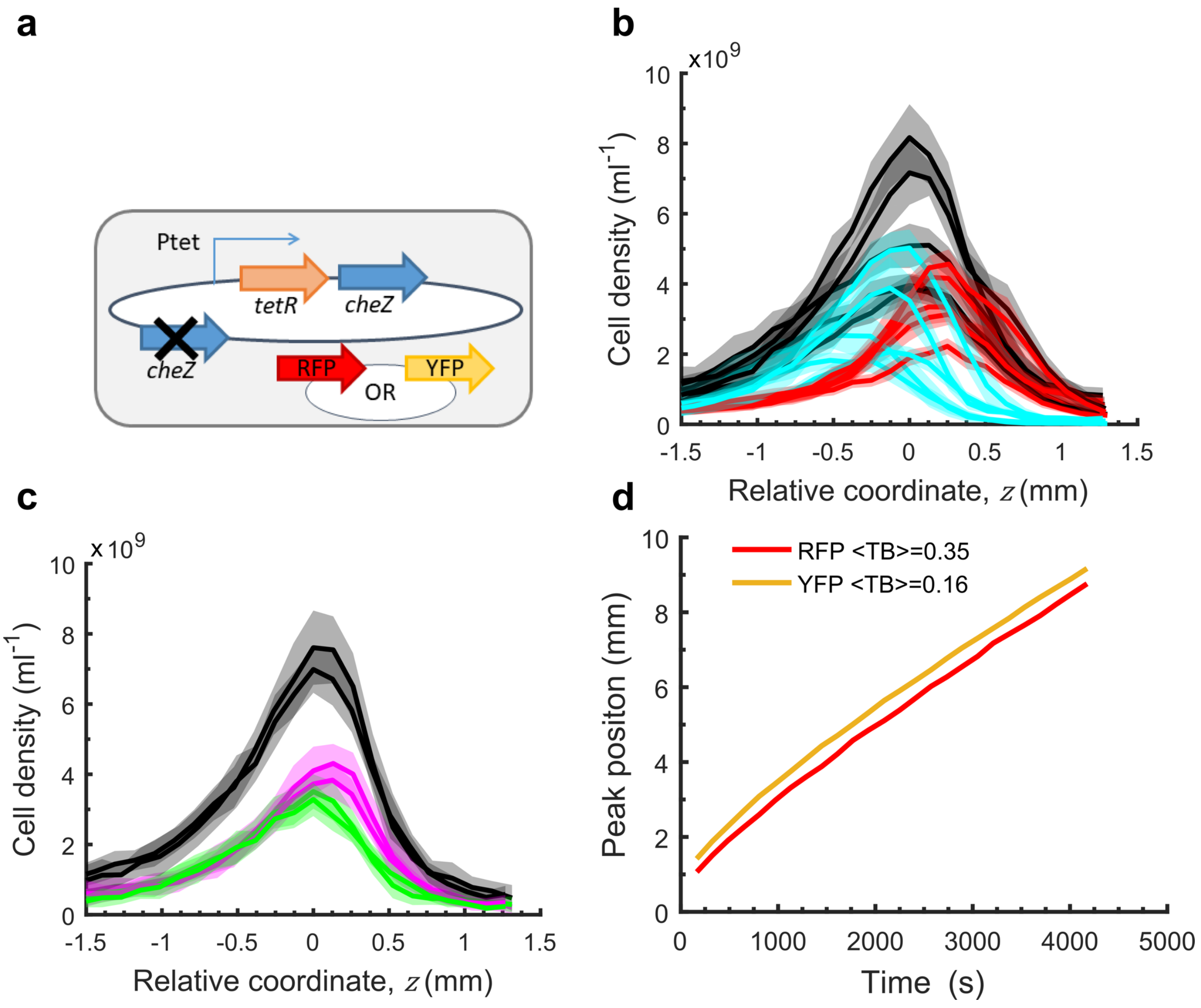

Supplementary Figure 9 | Spontaneous ordering of phenotypes in a traveling band.

a, The distribution of tumble bias in the population is controlled by manipulating the level of expression of the phosphatase CheZ, which deactivate the response regulator CheY. P*tet-tetR-cheZ* was introduced into the chromosome of *cheZ* knockout strain. The fluorescent protein is constitutively expressed from a plasmid. CheZ dephosphorylates the response regulator CheY, which, when phosphorylated, induces the motors to switch and the cells to tumble. Thus, low CheZ results in higher CheY-P and more tumbling, and vice versa. **b,** Five replicate experiments for 2 and 40 ng ml^{-1} induction of aTc. Cell density profiles for each population are shown in cyan and red, respectively while the total cell density is shown in black. Line: mean over n=34, 31, 19, 23 and 19 measurements of the same band at different time points; time interval is 40 sec; shading: SD. **c,** Two replicate experiments for 4 and 6 ng/ml induction of aTc. Cell density profiles for each population are shown in green and magenta, respectively. Lline: mean over 31 and 18 measurements of the same band; shading: SD. **d,** The peak distance between low and high TB populations stays constant over long time period. Cells expressing different level of CheZ were grown in the same conditions as for Fig. 4, mixed and introduced into a microfluidics device with the same geometry as described in Fig.1b but with a 3 cm long straight channel.

Supplementary References

1. Mesibov R, Adler J. Chemotaxis toward amino acids in Escherichia coli. *J Bacteriol* **112**, 315-326 (1972).

2. Yang Y*, et al.* Relation between chemotaxis and consumption of amino acids in bacteria. *Mol Microbiol* **96**, 1272-1282 (2015).
